# On the structure refinement of metal complexes against 3D electron diffraction data using multipolar scattering factors

**DOI:** 10.1107/S2052252524006730

**Published:** 2024-08-15

**Authors:** Laura Pacoste, Vladislav Mikhailovich Ignat’ev, Paulina Maria Dominiak, Xiaodong Zou

**Affiliations:** a Department of Materials and Environmental Chemistry Stockholm University Stockholm Sweden; b Biological and Chemical Research Center, Faculty of Chemistry University of Warsaw Warsaw Poland; Peking University, People’s Republic of China

**Keywords:** organometallic complexes, charge density distribution modelling, 3D electron diffraction, 3D ED, transferable aspherical atom model, TAAM, independent atom model limitations, electron crystallography, quantum crystallography

## Abstract

This study examines various methods for modelling the electron density and, thus, the electrostatic potential of an organometallic complex for use in crystal structure refinement against 3D electron diffraction (ED) data. It focuses on modelling the scattering factors of iron(III), considering the electron density distribution specific for coordination with organic linkers. We refined the structural model of the metal–organic complex, iron(III) acetyl­acetonate (FeAcAc), using both the independent atom model (IAM) and the transferable aspherical atom model (TAAM). TAAM refinement initially employed multipolar parameters from the MATTS databank for acetyl­acetonate, while iron was modelled with a spherical and neutral approach (TAAM ligand). Later, custom-made TAAM scattering factors for Fe—O coordination were derived from DFT calculations [TAAM-ligand-Fe(III)]. Our findings show that, in this compound, the TAAM scattering factor corresponding to Fe^3+^ has a lower scattering amplitude than the Fe^3+^ charged scattering factor described by IAM. When using scattering factors corresponding to the oxidation state of iron, IAM inaccurately represents electrostatic potential maps and overestimates the scattering potential of the iron. In addition, TAAM significantly improved the fitting of the model to the data, shown by improved *R*_1_ values, goodness-of-fit (GooF) and reduced noise in the Fourier difference map (based on the residual distribution analysis). For 3D ED, *R*_1_ values improved from 19.36% (IAM) to 17.44% (TAAM-ligand) and 17.49% (TAAM-ligand-Fe^3+^), and for single-crystal X-ray diffraction (SCXRD) from 3.82 to 2.03% and 1.98%, respectively. For 3D ED, the most significant *R*_1_ reductions occurred in the low-resolution region (8.65–2.00 Å), dropping from 20.19% (IAM) to 14.67% and 14.89% for TAAM-ligand and TAAM-ligand-Fe(III), respectively, with less improvement in high-resolution ranges (2.00–0.85 Å). This indicates that the major enhancements are due to better scattering modelling in low-resolution zones. Furthermore, when using TAAM instead of IAM, there was a noticeable improvement in the shape of the thermal ellipsoids, which more closely resembled those of an SCXRD-refined model. This study demonstrates the applicability of more sophisticated scattering factors to improve the refinement of metal–organic complexes against 3D ED data, suggesting the need for more accurate modelling methods and highlighting the potential of TAAM in examining the charge distribution of large molecular structures using 3D ED.

## Abstract 2

We apply for the first time the transferable aspherical atom model (TAAM) for the refinement of a metal complex structure against 3D ED data. Our results show that TAAM significantly outperforms the independent atom model (IAM) by more accurately depicting the electrostatic potential, particularly in low-resolution ranges. We found that using TAAM for organic ligands is more important than an accurate description of the metal centre in the refinement against 3D ED data.

## 1. Introduction

Three-dimensional electron diffraction (3D ED) has been extensively applied in studying a wide range of samples, from inorganic materials, small organic molecules and peptides to protein crystals (Gemmi *et al.*, 2019 ▸; Clabbers *et al.*, 2022 ▸; Danelius *et al.*, 2021 ▸; Liu *et al.*, 2017 ▸; Huang *et al.*, 2021 ▸; Clabbers & Xu, 2021 ▸). Its potential for structure determination is well established, offering the distinct advantage of analysing crystals in the sub-micrometre size-range, much smaller than those typically examined in conventional single-crystal X-ray diffraction (SCXRD).

In addition, 3D ED can provide valuable complementary information to SCXRD data, especially regarding the charge state of species within a crystal. This capability stems from the fact that electrons are charged and thus should be more sensitive to different charge states compared with X-rays. Yonekura *et al.* (2015 ▸) demonstrated the importance of this approach, showing how accounting for the negative and positive charges on titratable residues in proteins can improve structural refinement by reducing the deviation of the electrostatic potential model for the titratable side chains from experimental data. Differences in charge states are particularly evident from refinement against low-resolution data (*d*_min_ < 5 Å). The possibility of refining the charge states of metal ion co-factors in protein structures against 3D ED data is a topic of ongoing discussion (Yonekura *et al.*, 2015 ▸; Gallenito & Gonen, 2022 ▸; Yonekura & Maki-Yonekura, 2016 ▸; Blum *et al.*, 2021 ▸). Efforts have been made to refine charge states, but the precise impact of different charge states on the electrostatic potential map remains quantitatively undetermined.

Notably, electron scattering factors for charged atoms presented in the *International Tables for Crystallography Volume C* (Cowley *et al.*, 2006 ▸) are frequently cited in discussions about the enhanced ability of 3D ED to detect charge states. This often involves comparing the scattering factors of metal ions that have been assigned formal charges corresponding to their oxidation states (OSs) in specific complexes (Yonekura *et al.*, 2015 ▸; Gallenito & Gonen, 2022 ▸; Yonekura & Maki-Yonekura, 2016 ▸). However, applying this approach, assuming that the charge of a given metal ion directly aligns with its OS, will lead to an overestimation of the scattering amplitudes. The generic definition of the OS was reported by Karen *et al.* (2014 ▸) as ‘the OS of a bonded atom equals its charge after ionic approximation’, *i.e.* the OS would correspond to the charge of a species if all bonds were purely ionic. However, this assumption is not valid in coordination complexes, such as those involving metal ions in MOFs or as co-factors in proteins (Maglio *et al.*, 2012 ▸; Lepetit *et al.*, 2016 ▸; Kubin *et al.*, 2018 ▸). This is further highlighted by the fact that such assumptions generate negative Fourier difference peaks around the metal ion centre when charged scattering factors corresponding to the metal OS were applied, as demonstrated for iron(III) in heme catalase (Yonekura & Maki-Yonekura, 2016 ▸; Yonekura *et al.*, 2015 ▸), the magnesium(II) centre in β-galactosidase (Yonekura & Maki-Yonekura, 2016 ▸) and the zinc(II)-containing site of insulin (Blum *et al.*, 2021 ▸). To accurately assess the influence of charge on the electrostatic potential map, as revealed by ED data, it is essential to account for partial charges.

In addition to partial charges, it is also important to account for non-spherical characteristics of atoms in a molecule. The crystal field is never fully spherically symmetric, particularly when an atom forms strong directed bonds. Consequently, representing the crystal electron density (or electrostatic potential) using spherical atomic densities, as in the independent atom model (IAM), can lead to significant inaccuracies. A more accurate method involves parameterizing aspherical atomic electron densities and scattering factors with analytical functions. These kinds of models are known as multipole models (Dawson & Cochran, 1997 ▸; Kurki-Suonio, 1968 ▸; Stewart, 1969 ▸; Hirshfeld, 1971 ▸; Coppens *et al.*, 1971 ▸; Hansen & Coppens, 1978 ▸). The multipolar parameters can be refined against observed structure factors, for instance obtained through SCXRD data; thus, information about partial charges and electron density asphericity can be extracted directly from experimental data and used to characterize electronic properties of molecules and crystals (Macchi, 2020 ▸; Tolborg & Iversen, 2019 ▸). Multipolar refinement, however, drastically increases the number of parameters to be refined and demands high-quality data of sub-ångström resolution.

When such data are not available, one can still benefit from using enhanced scattering factors based on a multipolar model (*MM*). The multipolar parameters can be refined against theoretical structure factors obtained from quantum-chemical calculations for molecules or crystals under study. Alternatively, multipolar parameters can be transferred from *MM*s obtained for related molecules or crystals, following the observation that multipolar parameters for atoms in comparable chemical environments have similar values (Brock *et al.*, 1991 ▸). These improved scattering factors can then be used to refine atomic coordinates and atomic displacement parameters (ADPs).

Recognizing the similarity of multipolar parameters, databanks for ‘transferable aspherical atoms’ (pseudoatoms) have been established (Pichon-Pesme *et al.*, 1995 ▸), including the ‘databank of Multipolar Atom Types from Theory and Statistical clustering’ (MATTS) (Jha *et al.*, 2022 ▸; Rybicka *et al.*, 2022 ▸), superseding UBDB (Kumar *et al.*, 2019 ▸), ELMAM2 (Domagała *et al.*, 2012 ▸) and the Generalized Invariom Database (GID) (Dittrich *et al.*, 2013 ▸). These databanks are then used to create the transferable aspherical atom model (TAAM) (Bąk *et al.*, 2011 ▸) for the electron density of the molecule or crystal under study, and further to evaluate various electronic properties (Budniak *et al.*, 2022 ▸; Zarychta *et al.*, 2015 ▸) or to perform crystal structure refinements, *i.e.* TAAM refinements. In the case of SCXRD, it is well established that TAAM refinement significantly enhances the fit of the model to the data and improves atomic positions, especially for hydrogen atoms and anisotropic ADPs (Zarychta *et al.*, 2007 ▸; Jha *et al.*, 2020 ▸; Dittrich *et al.*, 2006 ▸). The results are comparable to Hirshfeld atom refinement (HAR), another non-spherical approach originating from quantum crystallography (Jayatilaka & Dittrich, 2008 ▸; Kleemiss *et al.*, 2021 ▸; Jha *et al.*, 2022 ▸). In the case of 3D ED, TAAM has already been shown to improve the fit of models of organic molecules to experimental data (Gruza *et al.*, 2020 ▸; Jha *et al.*, 2021 ▸). However, its application to structures containing metal ions and the impact of modelling metal ion electron density and electrostatic potential has not yet been explored.

Despite these advancements, most structures are still refined using IAM, which assumes independent, spherically averaged neutral atoms for electron scattering calculations. Although IAM based charged electron scattering factors have been modelled and are listed in the *International Tables of Crystallography Volume C*, they are not typically used in conventional refinement processes. We found that modelling using the charged scattering factors based on IAM led to worse refinement results, with an increased *R*_1_ value. Furthermore, small-molecule refinement software such as *olex2.refine* (Dolomanov *et al.*, 2009 ▸) defaults to neutral scattering factors, with the option for manual inclusion of the parametrization for the charged scattering factors. However, refinement software commonly employed in protein crystallography, such as *phenix.refine* (Afonine *et al.*, 2012 ▸) and *REFMAC5* (Murshudov *et al.*, 2011 ▸), do not support charged scattering factors for electrons.

Apart from questions regarding the appropriate scattering model, 3D ED additionally suffers from systematic errors arising from multiple scattering events, known as dynamical effects. These effects break the kinematical approximation, which assumes that each electron is scattered only once by the specimen. Consequently, agreement parameters (*R*_1_ values) range from 15 to 40%, much higher than typically observed in SCXRD. Despite these high *R*_1_ values, correct and unambiguous determination of atomic structures is still possible. Wang *et al.* (2018 ▸) demonstrated that 3D ED data can be used to determine atomic structures with an average deviation from the reference atom (ADRA) within the range 0.02–0.08 Å compared with SCXRD models.

Moreover, efforts to model the dynamical effects, such as the multi-slice method (Cowley & Moodie, 1957 ▸; Goodman & Moodie, 1974 ▸) and the Bloch wave method (Dudka *et al.*, 2008 ▸; Palatinus *et al.*, 2015 ▸, 2017 ▸), have enabled the refinement of hydrogen positions and the determination of chirality in various specimens (Klar *et al.*, 2023 ▸). However, these methods are computationally intensive and inhibit the merging of multiple datasets, limiting their accessibility. Thus, most structures are still refined using the kinematical approximation. Furthermore, it is crucial to establish the extent of the discrepancies between models and experimental data that arise from using scattering factors based on IAM versus those resulting from dynamical diffraction itself. Understanding this distinction will help to improve the refinement process and the interpretation of fine structural details in 3D ED data in cases where dynamical refinement is not accessible.

In this study, we collected 3D ED data on the organometallic complex iron(III) acetyl­acetonate (FeAcAc) to examine the impact of scattering factors based on different models (IAM and TAAM) on the kinematical crystal structure refinement and the fit between data and model. By studying this small metal–organic complex, we mimic the behaviour of metal ion centres in protein structures, while minimizing experimental challenges associated with protein crystallography, like limited resolution due to crystal imperfections, beam damage and diffuse solvent scattering. At the same time, the size of the unit cell allows for data in a resolution range that is particularly influenced by variations in charge states (<5 Å) (Yonekura *et al.*, 2015 ▸). Our findings indicate that organic ligands play a dominant role in the electrostatic potential modelling, while the influence of Fe(III) is considerably smaller than what IAM predicts. Thus, electrostatic potential cannot be accurately modelled using scattering factors based on IAM alone. To enhance the refinement process, models such as TAAM, which consider both the asphericity of atoms in the ligand and the atomic partial charges, including the partial charge of Fe(III), are necessary.

## 2. Methodology

### 2.1. Collection of 3D ED data

Iron(III) acetyl­acetonate crystalline powder (≥99.9% purity, FeAcAc) was purchased from Sigma–Aldrich. Before data collection, the sample was finely ground in a mortar, dispersed in water and applied onto a C-flat Holey Carbon Grid (CF-1.2/1.3, 300 mesh) for TEM. The grid was allowed to dry thoroughly at room temperature before being flash-frozen in liquid nitro­gen. 3D ED data were collected at 300 kV on a Titan Krios cryo-Transmission Electron Microscope (cryo-TEM) from ThermoFisher Scientific, equipped with a Ceta-D CMOS detector. The electron beam was configured using a spot size of 11, a C2 aperture of 20 µm and a beam diameter of 2 µm, resulting in an electron flux of 0.025 e Å^−2^ s^−1^. The 3D ED datasets were acquired over various tilt angles (ranging from −65 to +65°) with a tilt increment of 0.25° per frame and an exposure time of 0.5 s, leading to an average total fluence of 5.4 e Å^−2^ per dataset. All datasets were collected using a beam stop to minimize interference from the central spot that could affect the low-resolution reflections. Data processing, including reduction, scaling and merging, was carried out using *XDS* (Kabsch, 2010 ▸). Datasets were merged using *edtools* (Smeets *et al.*, 2022 ▸), where datasets were clustered depending on their relative correlation. The cluster that yielded the highest completeness (92.2%), highest CC_1/2_ correlation (99.5%) and lowest *R*_meas_ (22.8%) was further used for refinement. The datasets were deposited in Zenodo at https://doi.org/10.5281/zenodo.10470572.

### 2.2. Structure solution and refinement against 3D ED data

The structure was solved using *SHELXT* (Sheldrick, 2015 ▸) and later refined using *olex2.refine* from the *Olex2-1.5* (Dolomanov *et al.*, 2009 ▸) package that incorporates the possibility for TAAM refinement through the *NoSpherA2* implementation (Kleemiss *et al.*, 2021 ▸) via the *discambMATTS2tsc.exe* plugin (Chodkiewicz *et al.*, 2018 ▸; Jha *et al.*, 2020 ▸; Gruza *et al.*, 2020 ▸). For this study, three different sets of electron scattering factors were used: scattering factors based on IAM; TAAM scattering factors available through the MATTS data bank, which only considers the partial charges and asphericity of the organic ligand (TAAM-ligand); and custom-made TAAM scattering factors, which also take the Fe—O coordination into account [TAAM-ligand+Fe(III)]. All refinements were performed with anisotropic ADPs for non-hydrogen atoms. For hydrogen atoms, geometrical constraints were used with fixed bond lengths derived from neutron diffraction data as proposed by Allen & Bruno (2010 ▸). All refinements were performed against |*F*|^2^ and using the Levenberg–Marquardt algorithm. No extinction correction was used. The following weighting scheme was applied for the refinements:
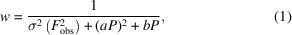
where *P* = (*F*_obs_^2^ + 2*F*_calc_^2^)/3. Optimized values for *a* and *b* were used for each refinement to reach a normal distribution of the residuals.

Crystallographic information files (CIFs) for all refinements are provided in the supporting information.

#### 2.2.1. IAM refinement

The structure was initially solved and refined using neutral scattering factors derived from IAM. For charged scattering factors, both Fe^2+^ and Fe^3+^ scattering factors were used in the refinement together with O^0.5−^ partially charged oxygen. The scattering factor used for the partially charged oxygen was approximated by a linear combination of neutral and fully ionized oxygen O^−^, following the methodology described by Yonekura & Maki-Yonekura (2016 ▸). These were parametrized using a 4-Gaussian curve fitting, as required by *SHELXL* or *olex2.refine*, using nonlinear least-squares optimization with the Levenberg–Marquardt algorithm in Python, accessible via *Edtools* (Smeets *et al.*, 2022 ▸). For the scattering factors according to IAM, all scattering factors (except for O^0.5−^) are based on the UCLA 4-Gaussian fitting (Saha *et al.*, 2022 ▸), which are fitted to the neutral and charged scattering factors listed in the *International Tables for Crystallography Volume C* (Cowley *et al.*, 2006 ▸). The charged scattering factors used for the refinement are listed in Table S3.

Due to the large discrepancy between the model and the data for IAM, *olex2.refine* reached its limit in the weighting parameter optimizations and instead defaulted the values to *a* = 0.2 and *b* = 0. This was the case for all refinements using IAM. Weighting parameters were instead refined in *SHELXL* (Sheldrick, 2015 ▸), output in an .lst file, and used in the *olex2.refine* refinement to ensure comparability of the GooF parameters.

#### 2.2.2. TAAM refinement using MATTS databank

TAAM refinement was conducted to explore the impact of more precise modelling of atomic partial charges and asphericity of electrostatic potential, using the structure initially solved and refined with neutral scattering factors from IAM. As a preliminary step, we calculated TAAM scattering factors using the *discambMATTS2tsc* program, which is integrated with *Olex2-1.5*. This program is based on the *DiSCaMB* library (Chodkiewicz *et al.*, 2018 ▸) and has the capability of recognizing atom types and generate non-spherical atomic X-ray or electron scattering factors based on the Hansen–Coppens multipolar model (*MM*) (Hansen & Coppens, 1978 ▸) parametrized in the MATTS databank (Jha *et al.*, 2022 ▸). In this study, we used *discambMATTS2tsc* (version 2.101). The resulting scattering factors are output to a .tsc file and can then be utilized for TAAM refinement using *olex2.refine* and *NoSpherA2* within *Olex2-1.5* (Kleemiss *et al.*, 2021 ▸). Electron scattering factors are derived by transforming X-ray scattering factors, *f*^x^(*s*), initially produced by the *DiSCAMB library*, into electron scattering factors *f*^e^(*s*) using the Mott–Bethe formula (Peng, 1999 ▸; Mott & Massey, 1964 ▸; Bethe, 1930 ▸),
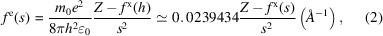
where *s* = sin(θ)/λ represents the reciprocal resolution, *m*_0_ and *e* are the rest mass and charge of the electron, *h* is Planck’s constant, ɛ_0_ is the vacuum permittivity, and *Z* is the atomic number.

The MATTS databank contains averaged multipolar parameters from families of chemically equivalent atoms. This imposed a limitation, because multipolar parameters describing coordinated Fe and O atoms were not available in the MATTS databank. Instead a hybrid IAM/TAAM procedure (Jha *et al.*, 2023 ▸) was used, in which the molecule was described as two separate parts: the Fe (part 1) and the organic acetyl­acetonate ligands (part 2). Although the MATTS databank provided multipolar parameters for the acetyl­acetonate molecule, it did not include parameters for Fe. Consequently, Fe was modelled using only the spherical and neutral components of the electrostatic potential, converted from electron density, which is described by the Clementi–Roetti Slater-type atomic wavefunction (Clementi & Roetti, 1974 ▸) in the *DiSCAMB* library. This is analogous to describing the atom using IAM, where the atom is considered spherical and neutral.

#### 2.2.3. TAAM refinement using custom-made TAAM parameters

Due to the absence of specific multipolar parameters for Fe and O in the MATTS databank relevant to the chemical environment of the FeAcAc molecule, we generated custom-made multipolar parameters [*MM*-ligand-Fe(III)] from the structure refined against the 3D ED data. The initial step involved calculating a molecular wavefunction from the structure of the FeAcAc complex refined with neutral scattering factors using IAM. The wavefunction was calculated using single-point static DFT calculations in *Gaussian16* (Frisch *et al.*, 2016 ▸) using the B3LYP method with a 6-31G** basis set and Fe(III) in high-spin configuration (Carlotto *et al.*, 2017 ▸). Using this wavefunction, valence-only X-ray structure factors were computed within an artificial cubic unit cell 30 Å in length, containing one FeAcAc complex. These structure factors were used for refining multipolar parameters with *XD2016* (Volkov *et al.*, 2016 ▸). We tested multiple combinations of the wavefunctions (Fe^0^, Fe^2+^, Fe^3+^), configurations and type of electrons associated with Fe allowed to be refined (Table S1 of the supporting information). The refinement that achieved the lowest *R* value and that was compatible with the *DiSCaMB* library was used for generating the custom-made TAAM scattering factors [TAAM-ligand-Fe(III)]. These multipolar parameters were refined using a Clementi–Roetti Slater-type atomic wavefunction for a neutral Fe atom (Clementi & Roetti, 1974 ▸) with a 3*d*^6^4*s*^2^ configuration, and only 3*d*^6^ electrons refined (4*s*^2^ electrons were frozen). The low *R*_1_ value (3.19%) relative to the wavefunction-derived structure factors indicated a strong correlation between the refined multipolar parameters and the theoretical wavefunction (Table S1).

Finally, these custom-made multipolar parameters were used to generate a custom-made databank, where the multipolar parameters were averaged for particular atom types. This databank was subsequently used to produce new TAAM scattering factors [TAAM-ligand+Fe(III)] using *discambMATTS2tsc* (version 3.006), following the same procedure as with MATTS databank parameters described in the previous section. This generated a .tsc file for use in subsequent refinements in *olex2.refine* through the *NoSpherA2* implementation (Kleemiss *et al.*, 2021 ▸).

### 2.3. Refinement against X-ray diffraction data

X-ray diffraction data of FeAcAc collected at 100 K with Cu *K*α radiation were obtained through the Cambridge Structural Database (CSD code: 1499479). Synthesis, crystallization procedure and details of the data collection are described by Arslan *et al.* (2017 ▸). Refinements using IAM, TAAM-ligand and TAAM-ligand+Fe(III) scattering factors were performed in the same way as for the 3D ED data, with the same atom labels as for the 3D ED refined model, optimized weighting parameters (*a* and *b*) and geometrical constrains for hydrogen atoms with fixed bond lengths derived from neutron diffraction data. However, the extinction correction parameter was refined as it was used in the original refinement in the CIF deposited by the authors. The resolution of the data was adjusted to *d*_min_ = 0.85 Å from the original 0.83 Å to maintain consistency with the resolution of the 3D ED data. CIFs for all refinements together with corresponding .tsc files (where relevant) are provided in the supporting information.

### 2.4. Generating Fourier difference maps between wavefunction, TAAM and spherical approximation

Fourier difference maps for electron density were generated using *XDFOU* from the *XD2016* suite (Volkov *et al.*, 2016 ▸) to compare X-ray structure factors from the wavefunction with those computed multipolar parameters [*MM*-ligand+Fe(III)] or a spherical neutral model from Table 6.1.1.4 of the *International Tables for Crystallography Volume C* (Maslen *et al.*, 2006 ▸). This helped us to visualize the differences between the wavefunction, multipolar and spherical neutral models. We also calculated electron structure factors from both refined multipolar parameters and the spherical neutral model [Table 4.3.2.3 of the *International Tables for Crystallography Volume C* (Cowley *et al.*, 2006 ▸)] to produce a Fourier difference map for electrostatic potential. The utility program from the *DiSCaMB* library (developer version) was used to produce xd.fou files containing appropriate structure factors read by *XDFOU*.

### 2.5. Bader charge analysis

To investigate the atomic charges surrounding the atoms in the FeAcAc molecule, Bader charges, also referred to as atoms-in-molecules (AIM) charges, were computed. Bader analysis utilizes electron density to estimate the net charge on each atom. It partitions a spatial function, like electron density, into Bader volumes at points where the gradient of electron density [∇ρ(*r*)] is zero. These zero flux points define distinct regions (Bader volumes, atomic basins) for each atom, and the integral of density within these volumes, is defined as the net charge of an atom (Bader, 1985 ▸; Posysaev *et al.*, 2019 ▸).

Various methods are available for calculating atomic charges in molecules, most of which require access to the wavefunction, such as Mulliken population analysis (Mulliken, 1955 ▸), Löwdin charge (Thompson *et al.*, 2002 ▸) and Natural Population Analysis (Reed *et al.*, 1985 ▸). Others utilize a refined reference proatom or ion, including Hirshfeld (Hirshfeld, 1977 ▸; Finzel *et al.*, 2015 ▸; Verstraelen *et al.*, 2013 ▸) and DDEC methods (Manz & Limas, 2016 ▸; Manz & Sholl, 2012 ▸). Bader charge analysis was chosen due to its application to electron densities from various sources, enabling effective comparisons between Bader charges from multipolar modelling and those derived directly from wavefunction. As the TAAM scattering factors are derived from the *MM*, Bader charges serve as a convenient tool for assessing the representativeness of TAAM scattering factors in capturing wavefunction behaviour and evaluating the electron density within the molecule.

Bader charges were calculated using the *Multiwfn* software (Lu & Chen, 2012 ▸) based on both the wavefunction and the electron density produced by the *MM*. The electron density for the *MM* was generated using *XDPROP* in the *XD2016* suite (Volkov *et al.*, 2016 ▸), within a cubic volume of 13.5 × 13.5 × 13.5 Å and a grid spacing of 0.03 Å. The same grid spacing was used for calculating the Bader charges from the wavefunction to ensure consistent analysis.

## 3. Results and discussion

### 3.1. IAM charged scattering factors severely overestimate scattering of charged species

To ensure comprehensive data coverage, three datasets were merged, resulting in an overall data completeness of 92.2% and a resolution of *d*_min_ = 0.85 Å. The acquisition statistics for this final combined dataset are detailed in Table 1 ▸. The crystal structure was determined in the space group *Pbca*, with unit-cell parameters deviating by 1% from those previously reported for this molecule from SCXRD data (Arslan *et al.*, 2017 ▸).

**Figure 1:**
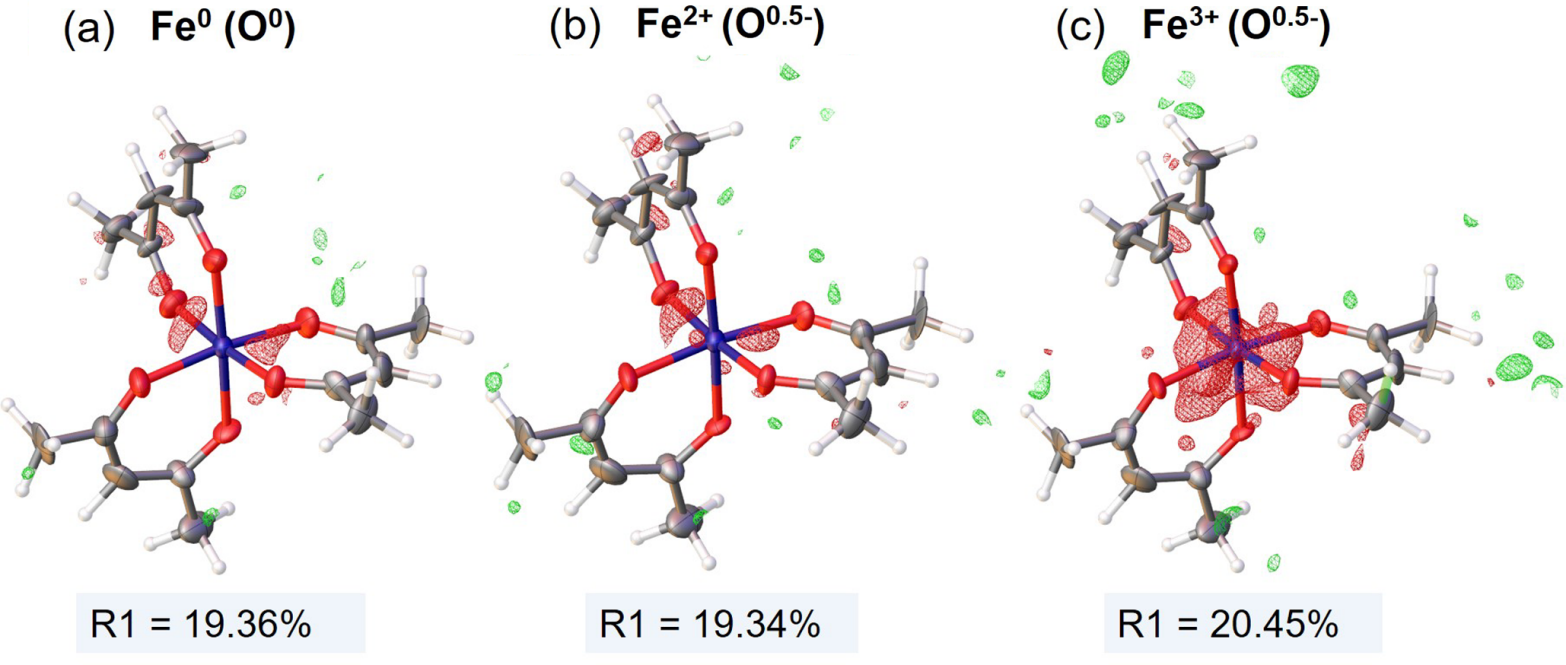
*F*_obs_ − *F*_calc_ Fourier difference electrostatic potential map (level 0.85 Å^−2^) for IAM refinement with (*a*) neutral iron and oxygen scattering factors, (*b*) +2 charged iron (Fe^2+^) and −0.5 partially charged oxygen scattering factors, and (*c*) +3 charged iron (Fe^3+^) and −0.5 partially charged oxygen scattering factors. Carbon and hydrogen atoms were always assigned neutral scattering factors. Ellipsoids for all non-hydrogen atoms are displayed with 50% probability level and hydrogens are shown with fixed radii. Figures were made in *Olex2*. *F*_obs_ – experimental structure factors, *F*_calc_ – structure factors computed from refined model. Contours colour code: green – positive, red – negative.

Initial refinement used neutral scattering factors for all species, resulting in a model with an *R*_1_ value of 19.36%. This model exhibited negative Fourier difference peaks between the oxygen of the acetylacetonate molecules and the Fe [Fig. 1 ▸(*a*)]. To test the accuracy of the Fe^3+^ charged scattering factor in representing the observed electrostatic potential, the model was further refined using this factor for Fe^3+^ along with a scattering factor of partially charged oxygen O^0.5−^. The assignment of the partial charge of oxygen was to balance the net molecular charge of −1 in the acetylacetonate molecule. Additionally, the refinement process included the Fe^2+^ charged scattering factor to assess if the charge representing the Fe(II) oxidation state provided a better fit. However, the charged scattering factors resulted in a poorer fit compared with the neutral factors, as shown in Figs. 1 ▸(*a*) and 1 ▸(*b*), with either unchanged (19.35% for Fe^2+^) or increased (20.45% for Fe^3+^) *R*_1_ values and large negative Fourier differences around the iron. These findings indicate that using the charged scattering factors and the ionic approximation as a representation of the Fe(III) charge state do not accurately reflect the experimental electrostatic potential map. Instead, assigning a +2 or +3 charge to the iron centre significantly overestimates its scattering potential, even after considering balancing charges from the oxygen atoms.

### 3.2. TAAM scattering factors significantly improves the model accuracy of organic ligands

Due to the limitations in accurate modelling of electrostatic potential with IAM, TAAM refinement was initially employed utilizing multipolar parameters from the MATTS databank (TAAM-ligand). However, the MATTS databank lacked specific parameters for Fe and O in coordination. Therefore, the molecule was modelled in two parts: the Fe and the organic acetyl­acetonate. Though available multipolar parameters were used for acetyl­acetonate, Fe was modelled using only spherical and neutral components of electrostatic potential, similar to the IAM approach where atoms are considered spherical and neutral (Clementi & Roetti, 1974 ▸). Due to the absence of multipolar parameters in the MATTS databank relevant to the Fe—O coordination in the FeAcAc molecule, custom-made TAAM scattering factors were generated from DFT calculations [TAAM-ligand+Fe(III)].

**Figure 2:**
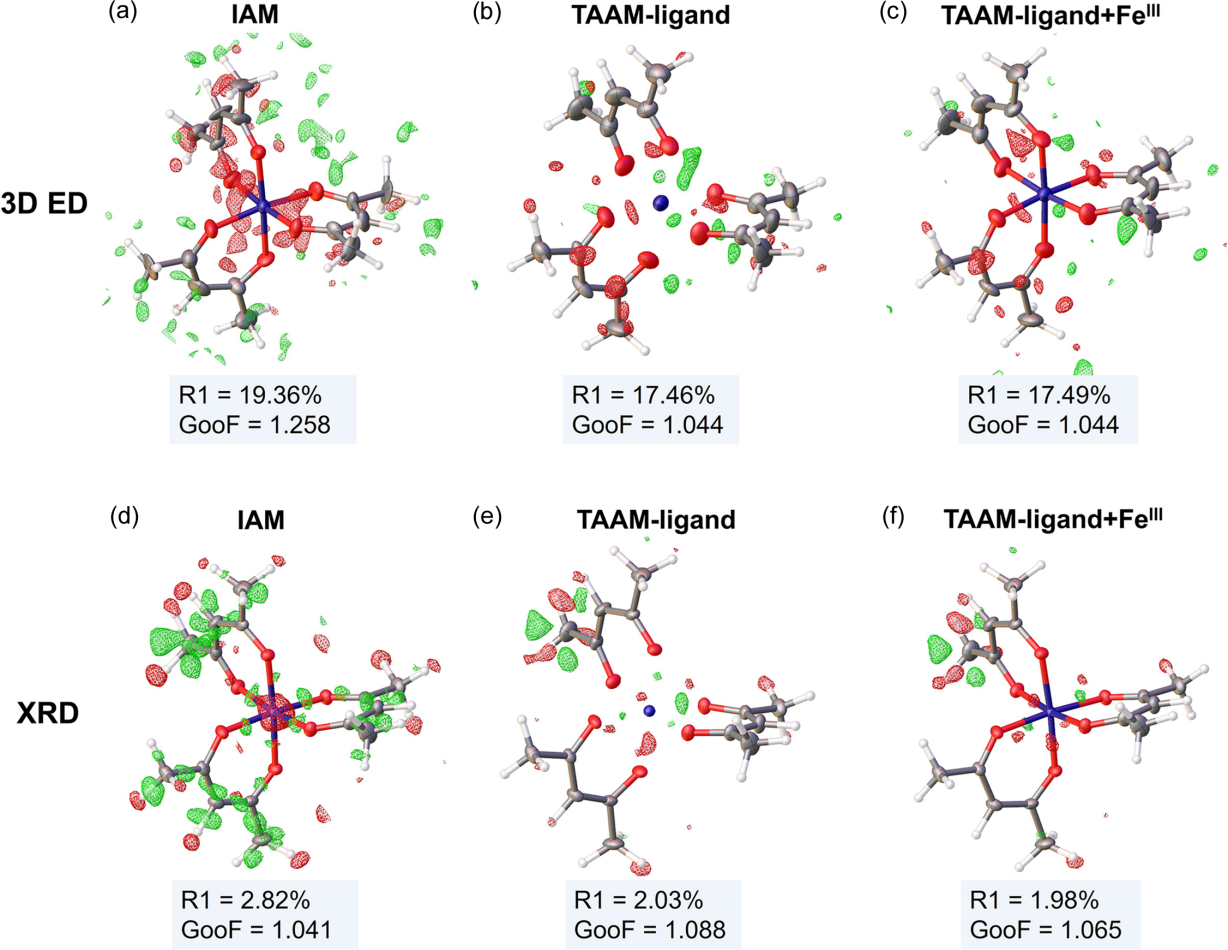
*F*_obs_ − *F*_calc_ difference maps from refinement against (*a*)–(*c*) 3D ED data (difference electrostatic potential map level 0.7 Å^−2^) and (*d*)–(*f*) SCXRD data (difference electron density map level 0.15 e Å^−3^), using neutral IAM scattering factors, TAAM-ligand scattering factors and custom-made TAAM-ligand+Fe(III) scattering factors. Structure as refined with TAAM-ligand scattering factors are displayed without Fe—O bonds to highlight that the models were refined in two parts with Fe (part 1) modelled with a spherical neutral approximation and acetylacetonate (part 2) refined with TAAM scattering factors. Ellipsoids for all non-hydrogen atoms are displayed with 50% probability level and hydrogen atoms are shown with fixed radii. Figures were made in *Olex2*. *F*_obs_ – experimental structure factors, *F*_calc_ – structure factors computed from refined model. Contours colour code: green – positive, red – negative.

**Figure 3:**
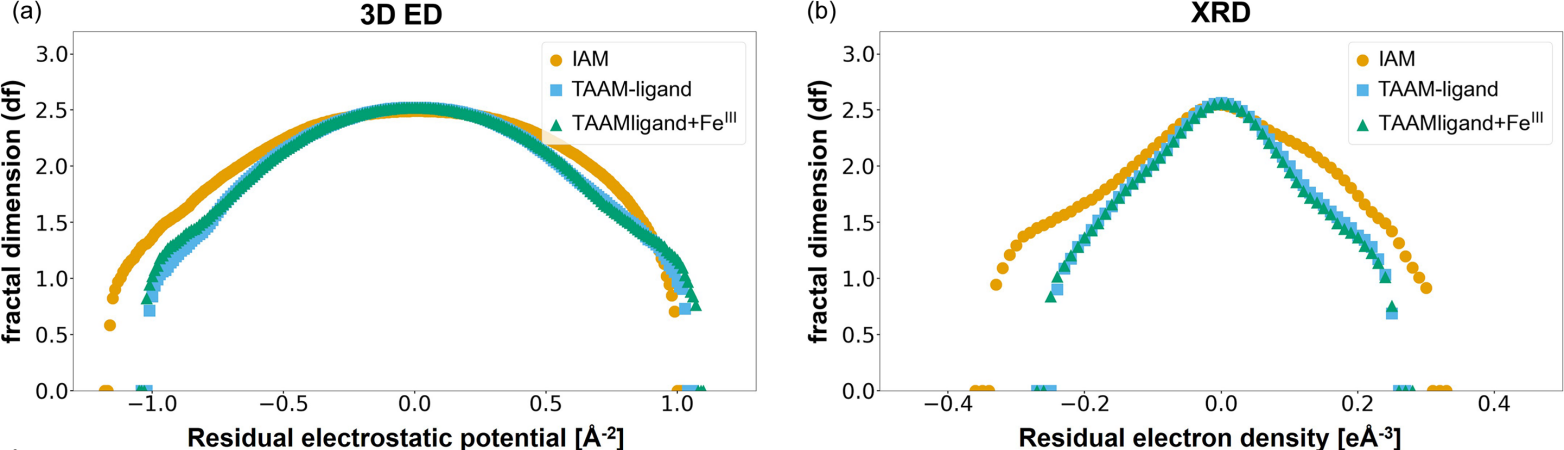
Comparison of fractal dimension plots computed for Fourier difference maps of the entire unit cell for refinement against (*a*) 3D ED data and (*b*) SCXRD data using IAM (yellow circle), TAAM-ligand (blue square) and custom-made TAAM-ligand+Fe(III) (green triangle) scattering factors. The fractal dimension plots were generated in *Olex2*.

Applying TAAM-ligand scattering factors to the organic ligands, while modelling the metal with a spherical neutral approximation, led to a notable reduction of about 1.9% units in the *R*_1_ value from 19.36% (IAM) to 17.45% (TAAM-ligand) and a decrease in the GooF from 1.258 to 1.044, as shown in Table 2 ▸. This suggests that the TAAM-ligand scattering factors significantly improved the fit of the model to the experimental 3D ED data. The use of custom-made TAAM-ligand+Fe(III) scattering factors, which included iron parametrization from DFT calculations, also resulted in a very similar *R*_1_ of 17.49% and a GooF of 1.044. Notably, inclusion of the metal ion in the *MM* did not significantly enhance the model fit. Therefore, the observed improvement in the fit between IAM and TAAM is likely attributed to the enhanced modelling of the organic ligand. Additionally, TAAM-refined models generated a Fourier difference map with less noise, with reduced negative Fourier difference peaks around the oxygen and iron atoms [Figs. 2 ▸(*b*) and 2 ▸(*c*)]. Further analysis of the fractal dimensions plots (Meindl & Henn, 2007 ▸) computed for Fourier difference maps within the entire unit-cell volume indicated a better fit for both TAAM refined models, with the curve having a smaller width and being more symmetrical compared with IAM [Fig. 3 ▸(*a*)].

Furthermore, *olex2.refine* could not optimize the weighting parameters *a* and *b* for IAM due to a large discrepancy between the model and data. To ensure comparability of the GooF parameter across refinements, weighting parameters were refined in *SHELXL* and used in *olex2.refine*. For TAAM-refined models, an optimized weighting scheme was achievable directly through *olex2.refine*.

For comparison, the same analysis was conducted on SCXRD data for the identical compound (Arslan *et al.*, 2017 ▸). Unlike the 3D ED, the Fourier difference map, when using IAM scattering factors, revealed a positive difference along the conjugated carbonyl backbone [Fig. 2 ▸(*d*)]. This suggests an underestimation of electron density on the organic ligand. Additionally, the Fourier difference map showed a negative difference peak near the iron. When TAAM refinement, either TAAM-ligand or TAAM-ligand+Fe(III), was applied, the Fourier difference maps appeared less noisy [Fig. 2 ▸(*e*) and 2 ▸(*f*)]. This observation is further supported by the fractal dimension plots [Fig. 3 ▸(*b*)].

Although 3D ED data are subject to significant discrepancies between data and model due to dynamical effects, which disrupt the kinematic approximation and lead to high values of *R*_1_, the reduction in *R*_1_ value was comparable when contrasting IAM and TAAM with results of refinements against SCXRD data. In the case of 3D ED, the *R*_1_ value declined by 2.6% with the application of TAAM scattering factors, whereas for refinement against SCXRD data the reduction in the *R*_1_ value was 0.7–0.8%. This indicates that, despite limitations in 3D ED data, the improved models performs comparably well in enhancing model accuracy and refinement outcome for both 3D ED and SCXRD.

Despite the use of TAAM-ligand and TAAM-ligand+Fe(III) scattering factors resulting in better fit between the 3D ED data and the model, the atomic positions remained relatively unchanged, as shown in Table 2 ▸. We calculated the root mean square deviation (RMSD) for the models refined against 3D ED data and the TAAM-ligand+Fe(III) model refined against SCXRD for comparison. For the models refined against 3D ED data, the RMSD of the atomic coordinates was 0.20 Å for IAM, compared with 0.19 Å for both the TAAM-ligand and the TAAM-ligand+Fe(III) models, indicating that the atomic positions were virtually unchanged between these refinements. However, the shape of the thermal ellipsoids in the TAAM models more closely resembled those refined against SCXRD data, as shown by similar ratios of the maximum to minimum root-mean-square components (*R*_1_/*R*_3_, Table S2). The average *R*_1_/*R*_3_ ratio was improved from 2.23 for the IAM model to 1.79 and 1.85 for the TAAM-ligand and TAAM-ligand+Fe(III) models, respectively, against 3D ED data. The later ratios are significantly closer to the 1.52 ratio of the TAAM-ligand+Fe(III) model refined against SCXRD data. This correlation is further supported by the RMSD values between the models refined against 3D ED data and the TAAM-ligand+Fe(III) model refined against SCXRD as a reference. The RMSD of the *R*_1_/*R*_3_ ratios for the IAM model was 1.08, while it was reduced to 0.40 and 0.47 for the TAAM-ligand and TAAM-ligand+Fe(III) models, respectively (Table S2).

### 3.3. Improvement in TAAM refinement predominantly due to better modelling of low-resolution data

The influence of charge on 3D ED data is considered to mainly affect the low-resolution region of the data (Yonekura *et al.*, 2015 ▸). To investigate the resolution dependence of the improved fit between the IAM and TAAM refinements, we calculated the *R*_1_ values for different resolution shells (8.65–2.00 Å and 2.00–0.85 Å) while keeping all parameters fixed from the models refined against the entire resolution range. The largest drop in *R*_1_ value is observed for the low-resolution (8.65–2.00 Å) reflections (Table 3 ▸), which reduced from 20.19% (IAM) to 14.67% (TAAM-ligand) and to 14.89% [TAAM-ligand-Fe(III)]. In the high-resolution shell (2–0.85 Å) the improvement was much smaller, from 18.62% (IAM) to 18.24% (TAAM-ligand) and 18.23% [TAAM-ligand-Fe(III)]. The results confirm that the reduction in the *R*_1_ value is mainly attributed to the improved modelling of the scattering in the low-resolution region.

Many small-molecule structures crystallize in small unit cells, resulting in few reflections in the low-resolution region inspected (8.65–2.00 Å). However, large structures, like MOFs and protein structures, contain a significant amount of data in this resolution range. This suggests an advantage for these larger structures, especially proteins, in terms of charge state determination. Considering the pronounced charge on the organic ligand in the FeAcAc structure, this raises questions about the potential improvements in modelling a protein 3D ED structure if more advanced models, like TAAM refinement, were integrated into conventional protein refinement software such as *phenix.refine* (Afonine *et al.*, 2012 ▸) or *REFMAC5* (Murshudov *et al.*, 2011 ▸).

### 3.4. Asphericity is mainly allocated on organic ligands

To understand the observed differences between refinements using IAM and TAAM, we explored the Fourier difference maps for both electron density and electrostatic potential. These maps compare the electron densities or electrostatic potentials computed from the FeAcAc molecule wavefunction with the spherical neutral model (IAM) and the *MM* [*MM*-ligand-Fe(III)], assuming that atoms are static (ADPs equal to zero). The *MM*-ligand-Fe(III) was used for generating the TAAM-ligand-Fe(III) scattering factors discussed in previous sections.

**Figure 4:**
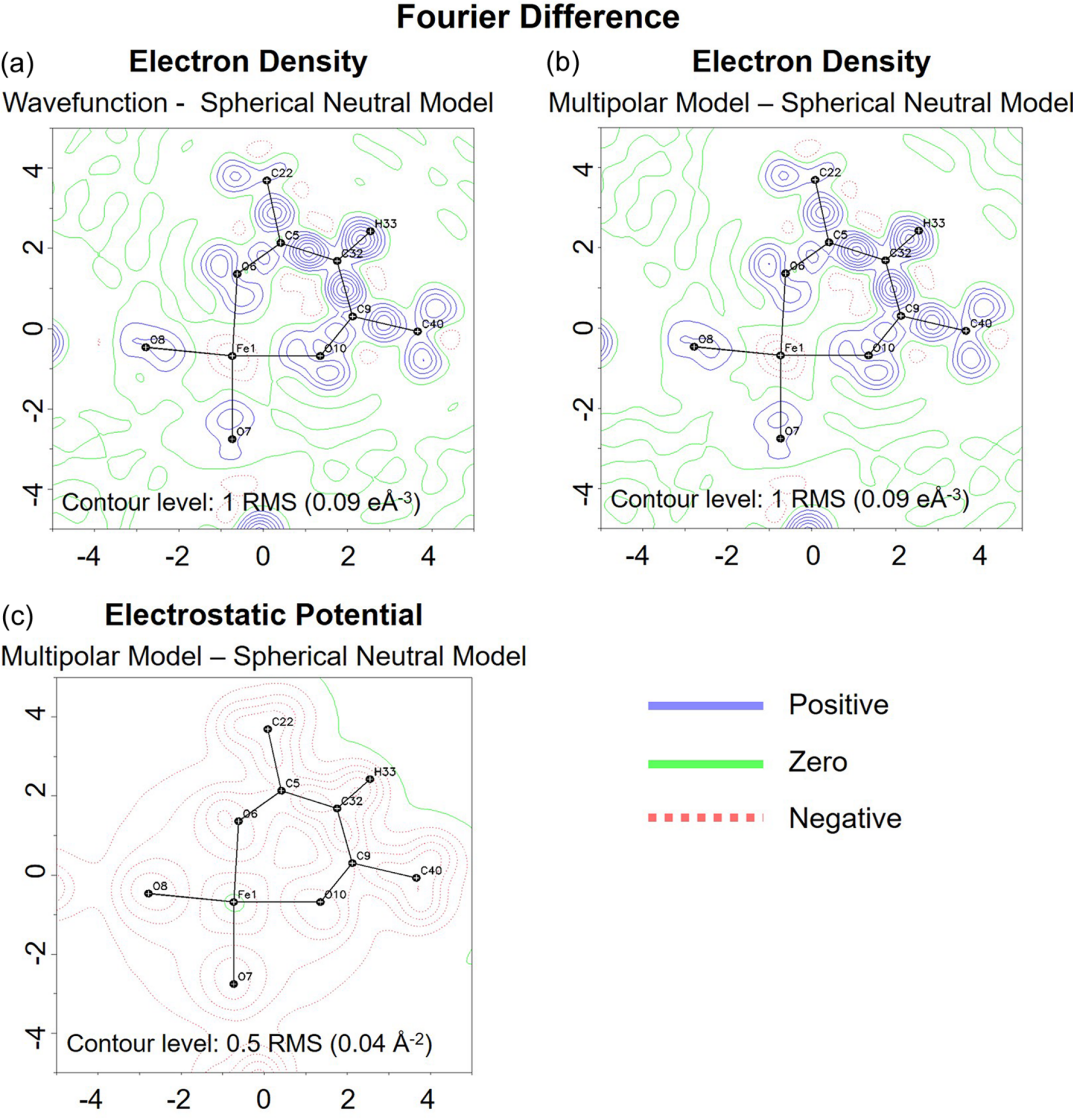
Comparison of the electron density Fourier difference maps obtained from (*a*) the wavefunction and spherical neutral model; (*b*) the wavefunction and the refined *MM* [*MM*-ligand-Fe(III)], contour interval ±1 RMS (0.09 e Å^−3^); and (*c*) the electrostatic potential Fourier difference map obtained from the refined *MM* [*MM*-ligand-Fe(III)] and a spherical neutral model, contour interval ±0.5 RMS (0.04 Å^−2^). The maps are computed at 0.85 Å resolution to ensure comparability with the experimental data. Contours colour code: green – zero, blue – positive, red – negative.

On examining the Fourier difference map between the wavefunction and the spherical neutral model [Fig. 4 ▸(*a*)], it becomes evident that asphericity in electron density predominantly resides within the organic part of the molecule, rather than the iron component. The *MM*-ligand-Fe(III) model refined against structure factors computed from the wavefunction very well reproduce this observation, as shown by the close resemblance between the Fourier difference map for the *MM*-ligand+Fe(III) and spherical neutral models [Fig. 4 ▸(*b*)] and the Fourier difference map for the wavefunction and the spherical neutral model [Fig. 4 ▸(*a*)].

A similar trend in distribution of aspherical features in the FeAcAc complex is observed for the electrostatic potential [Fig. 4 ▸(*c*)]. Moreover, these difference maps highlight that the electrostatic potential is more diffused compared with electron density. This implies that differences in the 3D ED electrostatic potential map, influenced by partial charges, are distributed over a larger volume of the unit cell than in SCXRD data, thereby more significantly affecting data of lower resolution.

Further analysis of the TAAM-ligand+Fe(III) [resulting from *MM*-ligand-Fe(III)] revealed that the Fe scattering factor is dominated by the spherical component which includes partial charge, while oxygen scattering factor has a significant aspherical contribution arising from higher multipole moments (Figs. S1 and S2 of the supporting information).

### 3.5. Bader charges explain the scattering of Fe(III)

On refining with IAM using scattering factors that correspond to the assigned oxidation state of iron, it became evident that the atomic charge in the experimental data did not align with the expected oxidation state (Section 3.1). Refinement against 3D ED data with TAAM scattering factors suggested that the iron is more accurately represented by a neutral, spherical shape, rather than possessing a +3 charge. This finding is logical when considering that the coordination bonds between Fe and O should not be viewed as purely ionic, but rather as involving a distribution of partial charges.

To further analyse the atomic charges derived from the wavefunction and the *MM*, we computed the Bader charges, also known as AIM charges. This analysis revealed that the Bader charge on the iron atom is +1.86, deviating from the anticipated +3, as shown in Table 4 ▸. Correspondingly, the refined *MM*-ligand-Fe(III) yielded a similar Bader charge of +1.66 for the iron atom. Intriguingly, the carbonyl oxygen atoms exhibited an average Bader charge of −1.20, contrasting with the expected −0.5. This disparity is balanced by a positive partial charge of +0.83 on the carbonyl carbons constituting the conjugated change. The atomic charges calculated from the wavefunction through DFT calculations and those from the *MM*-ligand-Fe(III) closely correspond, with only a slight discrepancy noted for the iron atom.

These findings emphasize the crucial role of electron density and electrostatic potential of the organic ligand, especially regarding the oxygen atoms and the conjugated chain, in explaining the discrepancy between the IAM and the experimental data. Noteworthy is also that high partial charges are mainly located on the oxygen and carbonyl carbon, while further away from the iron centre of the molecules, the carbons are neutral. This indicates that modelling the iron coordinating atoms and the atoms directly connecting to them is already sufficient for describing electrostatic potential and improving upon IAM.

In order to assess the impact of the coordination environment, we compared IAM scattering factors with custom-made TAAM-ligand+Fe(III) scattering factors more closely. The TAAM-ligand+Fe(III) scattering factors were plotted, taking into account only the spherical contribution to the multipole model of electron density (Clementi & Roetti, 1974 ▸), where core and valence spherical electron densities are treated separately. The ionic scattering factors from the TAAM-ligand+Fe(III) model were calculated with the valence electron scattering factors scaled to match the actual number of valence electrons (*P*_val_) and adequately modified by the value of the expansion–contraction parameter (κ): *f* = *f*_core_ + *P*_val_*f*_val_(κ), where *f*_core_ and *f*_val_ represent the scattering factor contributions from core and valence spherical electron densities, respectively.

**Figure 5:**
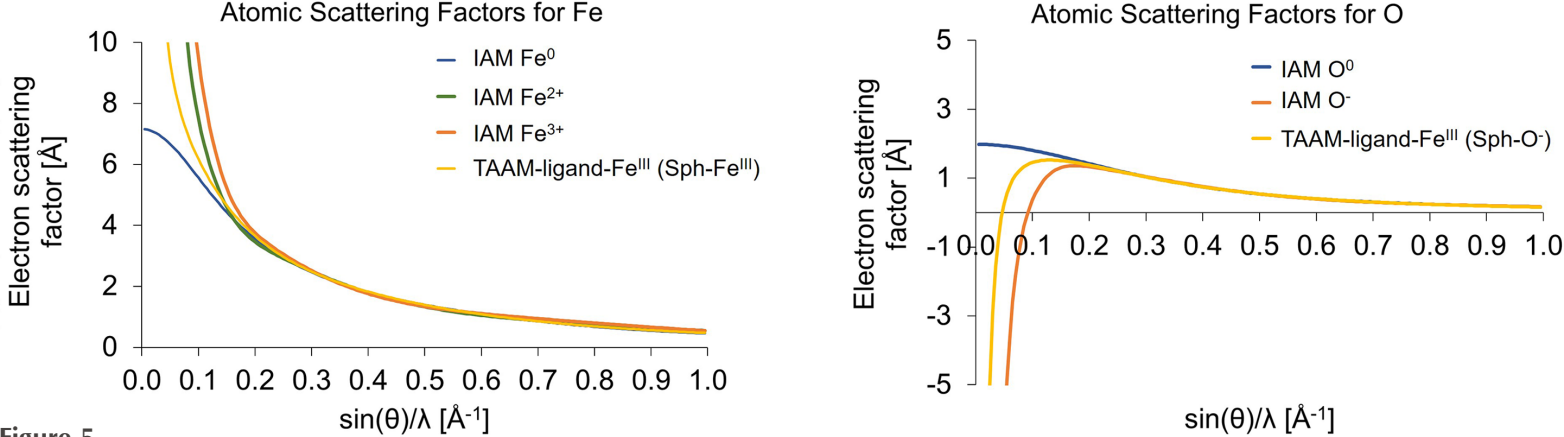
Atomic scattering factors for (*a*) iron and (*b*) oxygen from IAM (various charge states) and for the spherical component of the TAAM-ligand+Fe(III) model.

Plotting the spherical contribution to the TAAM-ligand+Fe(III) model alongside IAM atomic scattering factors [Fig. 5 ▸(*a*)] revealed that the TAAM-ligand+Fe(III) parameters, based on a molecular wavefunction calculation assuming iron in a +3 OS, produce an electron scattering factor for Fe with values that lie between the IAM scattering factors for Fe^0^ and Fe^2+^. Notably, the Fe^3+^ scattering factor from IAM overestimates the scattering amplitude up to 0.20 Å^−1^ (*d* = 2.5 Å). TAAM-ligand+Fe(III) accounts for both partial charges and the surrounding environment, significantly reducing the additional scattering power that IAM predicts for charged species. This elucidates why IAM frequently results in a marked overestimation of electron scattering for positively charged species, and partially explains why neutral scattering factors often yield a better fit to the data compared with the charged scattering factors from IAM. If partial charges are to be considered, it is important to model these using aspherical scattering factors that better account for the actual electron density and electrostatic potential reflected in the data.

A similar pattern can be observed with oxygen [Fig. 5 ▸(*b*)], where the O^−^ scattering factor based on IAM tends to overestimate the negative contribution from oxygen. This could be partly attributed to the analysis, focusing only on the spherical contribution from the TAAM-ligand+Fe(III) model, neglecting the higher moments in multipole expansion. However, the aspherical contribution to the electron scattering factor of oxygen in the TAAM-ligand+Fe(III) model is relevant only at resolutions of 0.02–0.07 Å^−1^ (*d* = 25–7.14 Å) (Fig. S2). Hence, this does not sufficiently explain the pronounced discrepancy between IAM (O^−^) and TAAM-ligand+Fe(III)(Sph-O^−^) observed in the range 0.07–0.20 Å^−1^ (*d* = 7.14–2.5 Å), where the aspherical contribution accounts for only 1% at these resolutions. This suggests that the IAM electron scattering factor for O^−^ does indeed overestimate the negative contribution from oxygen in these resolution ranges.

## 4. Conclusions

Our research reveals that modelling charged species using IAM, particularly when assigning formal charges corresponding to the the oxidation state of iron, fail to reflect the true experimental electrostatic potential map. Instead, charged scattering factors based on IAM tend to overestimate the scattering potential of iron, despite accounting for the balancing charges from oxygen atoms. In contrast, TAAM, which considers partial charges and asphericity, demonstrates more accurate results. This is evident from the significant improvements in *R*_1_ and GooF. For example, in 3D ED data, the *R*_1_ value improved from 19.36% with IAM to 17.44% for TAAM-ligand and 17.49% for TAAM-ligand-Fe(III). Similarly, in SCXRD, the *R*_1_ value decreased from 3.82% with IAM to 2.03% for TAAM-ligand and 1.98% for TAAM-ligand-Fe(III). This demonstrates that, despite the limitations caused by multiple scattering effects in 3D ED data, the improved scattering models effectively enhance the fit of the model to the data at a level comparable to SCXRD.

The influence of charge on 3D ED data is particularly pronounced in the low-resolution region of the data. Our investigation into the resolution dependency of the improved fit between IAM and TAAM refinements showed that the largest drop in *R*_1_ value occurs in the low-resolution reflections. Specifically, the *R*_1_ value for low-resolution data (8.65–2.00 Å) reduced from 20.19% (IAM) to 14.67% (TAAM-ligand) and 14.89% [TAAM-ligand-Fe(III)]. In contrast, in the high-resolution shell (2.00–0.85 Å), the improvement was much less, with *R*_1_ values changing from 18.62% (IAM) to 18.24% (TAAM-ligand) and 18.23% [TAAM-ligand-Fe(III)]. This confirms that the main contribution to the reduction in *R*_1_ value is the improved modelling of scattering in the low-resolution area.

Furthermore, our analysis suggests that accurate modelling of organic ligands is more crucial for fitting accuracy than detailed metal ion representation. The asphericity, or deviation from spherical symmetry in electron density, is mainly observed within organic ligands, rather than within the metal ion, as evident by the Fourier difference map between the wavefunction and a spherical representation of the model. This explains the apparent accuracy of neutral IAM scattering factors for metal ions. Bader charge analysis further confirms that the metal ion charge is considerably smaller than its formal oxidation state, highlighting the need for more sophisticated scattering factors in 3D ED data refinement to accurately model electrostatic potential.

In summary, using charged scattering factors based on IAM significantly worsens the fit between model and data, compared with neutral scattering factors, when refining against 3D ED data. Furthermore, our findings indicate that conventional models like IAM should be reconsidered, particularly given the promising outcomes with TAAM refinement against 3D ED data. This suggests a shift towards more sophisticated modelling techniques that more accurately represent complex molecular structures, especially for accurately capturing electron density and electrostatic potential of larger molecules. However, further studies and validations are required to fully confirm these advancements. We anticipate that advanced models such as TAAM will eventually be incorporated into standard protein refinement software like *phenix.refine* or *REFMAC5*, potentially enhancing the modelling of protein 3D ED structures.

## Supplementary Material

Crystal structure: contains datablock(s) 3DED_IAM_neutral, 3DED_IAM_charged_Fe2, 3DED_IAM_charged_Fe3, 3DED_TAAM-ligand, 3DED_TAAM-ligand-FeIII, XRD_IAM_neutral, XRD_TAAM-ligand, XRD_TAAM-ligand-FeIII. DOI: 10.1107/S2052252524006730/jf5002sup1.cif

.tsc file used for refinement against 3DED data containing the TAAM-ligand-FeIII scattering factors. DOI: 10.1107/S2052252524006730/jf5002sup2.txt

.tsc file used for refinement against XRD data containing the TAAM-ligand-FeIII scattering factors. DOI: 10.1107/S2052252524006730/jf5002sup3.txt

Supporting tables and figures. DOI: 10.1107/S2052252524006730/jf5002sup4.pdf

CCDC references: 2374930, 2374931, 2374932, 2374933, 2374934, 2374935, 2374936, 2374937

## Figures and Tables

**Table 1:** 3D ED data acquisition statistics

Temperature (K)	77
Space group	*Pbca*
*a*, *b*, *c* (Å)†	15.35 (5), 13.56 (7), 16.50 (5)
No. of crystals merged	3
Accumulated fluence per dataset (e Å^−2^)	5.5, 5.0, 5.8
Resolution (Å)‡	8.65–0.85 (0.87–0.85)
*R*_meas_ (%)‡	23 (136)
Mean *I*/σ(*I*) ‡	8.41(1.97)
CC1/2‡	99.7(65.4)
Completeness (%)‡	92.2(87.4)
Redundancy‡	18.6 (18.4)

† Values in parentheses represents the standard error for the unit-cell parameters.

‡ Values in parentheses represent values for the highest-resolution shell.

**Table 2:** Refinement parameters for models refined against 3D ED and SCXRD data using IAM scattering factors, TAAM-ligand scattering factors and custom-made TAAM-ligand+Fe(III) scattering factors

	3D ED			SCXRD		
Refinement parameter	IAM	TAAM-ligand	TAAM-ligand-Fe(III)	IAM	TAAM-ligand	TAAM-ligand-Fe(III)
No. of reflections†	2007(2687)	2007(2687)	2007(2687)	2611 (2821)	2611 (2821)	2611 (2821)
No. of parameters refined	205	205	205	206	206	206
*R*_1_ (%)†	19.36(22.79)	17.46(20.98)	17.49(21.01)	2.82(3.05)	2.03(2.27)	1.98(2.21)
GooF	1.258	1.044	1.044	1.0412	1.088	1.0649
Max. difference peak	0.924 Å^−2^	1.012 Å^−2^	1.092 Å^−2^	0.2958 e Å^−3^	0.2368 e Å^−3^	0.2412 e Å^−3^
Min. difference peak	−1.0768 Å^−2^	−0.995 Å^−2^	−1.015 Å^−2^	−0.3151 e Å^−3^	−0.2337 e Å^−3^	−0.23 e Å^−3^
Optimized weights (*a*, *b*)	0.2155, 2.1700‡	0.1493, 4.7933	0.1483, 5.0209	0.0437, 2.2044	0.0200, 0.9656	0.0187, 0.9913
RMSD§						
Atoms (Å)	0.20	0.19	0.19	0.0035	0.0019	N/A
Bonds (Å)	0.03	0.02	0.03	0.0044	0.0019	N/A

† Values given for reflections with *F*_o_ > [4σ(*F*_o_)] and values in parentheses report for all data.

‡ Weighting parameters (*a*, *b*) for the model refined with IAM could not be refined in *olex2.refine* and were therefore removed from refinement in *SHELXL*.

§ RMSDs, calculated for the fractional coordinates and bond distances of non-hydrogen atoms for each refinement. The model refined against SCXRD with TAAM-ligand+Fe(III) scattering factors was used as the reference model.

**Table 3:** *R*_1_ values and the number of reflections for refinements against data in different resolution shells

Locked refinement	All data	Low resolution	High resolution
Resolution (Å)	8.65–0.85	8.65–2	2.00–0.85
No. of reflections†	2007 (2687)	206 (208)	1801 (2478)
*R*_1_ values† (%)			
IAM	19.36 (22.79)	20.19 (20.16)	18.62 (23.05)
TAAM-ligand	17.46 (20.98)	14.67 (14.73)	18.24 (22.61)
TAAM-ligand-Fe(III)	17.49 (21.01)	14.89 (14.98)	18.23 (22.58)

† Values given for reflections with *F*_o_ > 4σ(*F*_o_) and values in parenthesis report for all data.

**Table 4:** Averages Bader charges (*e*) and standard deviations, derived from the wavefunction obtained from DFT calculations and the refined *MM*-ligand-Fe(III), for each atom type constituting the FeAcAc molecule

	Wavefunction		*MM*-ligand-Fe(III)	
Atom type	Average atomic charge	STD	Average atomic charge	STD
Fe	1.86	N/A	1.66	N/A
O	−1.19	0.02	−1.20	0.02
C (**C**=O)	0.83	0.04	0.83	0.04
C (**C**H_3_)	0.06	0.01	0.06	0.01
C (C—**C**H—C)	−0.07	0.01	−0.04	0.01
H (C**H**_3_)	0.01	0.01	0.02	0.01
H (C—**H**)	−0.02	0.00	0.00	0.00
