# Supplementary material for: On the structure refinement of metal complexes against 3D electron diffraction data using multipolar scattering factors
Source: IUCrJ. 2024 Aug 15;11(Pt 5):878–90. doi: 10.1107/S2052252524006730 (PMC11364031; doi:10.1107/S2052252524006730)
Supplement: Supplementary file 4 [file m-11-00878-sup4.pdf]

IUCrJ

Volume 11 (2024)

Supporting information for article:

On the structure refinement of metal complexes against 3D electron diffraction data using multipolar scattering factors

Laura Pacoste, Vladislav Ignatyev, Paulina Maria Dominiak and Xiaodong Zou

S1. Multipolar Model Refinements for Generating Custom Made TAAM Scattering Factors

Multiple combinations of parameters were investigated for refinement of the multipolar parameters used to generate the custom made TAAM scattering factors. These parameters included different Slater-type wavefunctions (Clementi & Roetti, 1974) representing Fe of different shape (Fe^0, Fe^{2+}, Fe^{3+}), electron configurations and electrons that were allowed to be refined (see Table S1). The multipolar refinements were performed in XD2016 (Volkov *et al.*, 2006) and evaluated on the R-value based on structure factors (R(F)), R-value for squared structure factors ($R(F^2)$) and weighted goodness-of-fit parameters (GOF), relative to the wavefunction derived structure factors. The GOF is defined as shown in Eq. S1:

$$GOF = \sqrt{\frac{\sum(\Delta F)^2}{(N_{ref} - N_{var})}} \qquad \text{Eq. S1}$$

where $\Delta F = (F_{obs} - k_1 F_{calc})$, F_{obs} represents the wavefunction derived structure factors, F_{calc} represents the structure factors calculated from the multipolar model, k_1 is a scale factor which in these refinements were set to 1.0 and not refined, and N_{ref} and N_{var} are the number of observations and number of independent variables in the least-square refinement.

Evaluation of the multipolar refinements revealed that refinement using a Slater-type wavefunction with neutral Fe shape (Fe^0) resulted in the lowest R(F) value of 3.18%, see run 3 in Table S1. The refinement outcomes were highly similar when refining only the 6d-orbital electrons and keeping the 4s electrons frozen (run 4, Table S1), with an R(F) value of 3.19% . Since the discambMATTS2tsc program, based on the DiSCaMB library (Chodkiewicz et al., 2018), assumes multipolar parameters refined with frozen $4s^2$ electrons, multipolar parameters from refinement run 4 were used for calculating the custom made TAAM scattering factors utilized for refinemet against 3D ED data.

Table S1 Parameters for the Fe atom and refinement outcome for multipolar refinements against wavefunction derived structure factors

All wavefunctions were calculated using the B3LYP method with 6-31G** basis set.

Run	Slater-type Wavefunction	Configuration	Refinement of 4s Electron	Refinement Outcome
1	Fe^{3+}	$3d^5$	N/A	R(F) = 0.0424
				$R(F^2)$ = 0.0215
				GOF = 0.0400
2	Fe^{2+}	$3d^6$	N/A	R(F) = 0.0379
				$R(F^2)$ = 0.0173
				GOF = 0.0337
3	Fe^0	$3d^64s^2$	free	R(F) = 0.0318
				$R(F^2)$ = 0.0100
				GOF = 0.0262
4	Fe^0	$3d^64s^2$	frozen	R(F) = 0.0319
				$R(F^2)$ = 0.0102
				GOF = 0.0264

S2. Thermal Ellipsoid Shape for IAM and TAAM Refinements

Evaluation of the root-mean-square components of the thermal ellipsoids R1, R2 and R3 (where R1 and R3 represents the longest and shorterst component), calculated as the square root of the principal mean square atomic displacement parameters (U1, U2, and U3).

Table S2 Ratio between maximum and minimum root-mean-square components (R1/R3) of the thermal ellipsoids from refinements against 3D ED and SCXRD data

Corresponding average RMSD values were calculated using the TAAM-ligand+Fe^{III} refined against SCXRD data as the reference model.

	R_1/R_3 ratio 3D ED			R_1/R_3 ratio SCXRD		
**Atom label**	**IAM**	**TAAM-ligand**	**TAAM-ligand+Fe^{III}**	**IAM**	**TAAM-ligand**	**TAAM-ligand+Fe^{III}**
Fe01	1.51	1.51	1.51	1.10	1.11	1.11
O001	1.93	1.98	2.14	1.42	1.66	1.50
O002	2.02	1.64	1.69	1.39	1.62	1.49
O003	1.71	1.57	1.58	1.41	1.64	1.49
O004	1.86	1.63	1.70	1.28	1.51	1.36
O005	1.63	1.42	1.42	1.33	1.54	1.41
O006	1.49	1.39	1.43	1.45	1.62	1.51
C001	1.43	1.37	1.35	1.45	1.50	1.44
C002	2.11	1.62	1.64	1.42	1.48	1.41
C003	1.70	1.74	1.80	1.51	1.43	1.42
C004	1.71	1.58	1.66	1.40	1.28	1.23
C005	1.55	1.48	1.53	1.46	1.48	1.39
C006	1.71	1.63	1.77	1.37	1.56	1.50
C007	3.77	2.67	2.81	1.71	1.81	1.76
C008	2.60	1.73	1.77	1.55	1.67	1.63
C009	2.77	2.06	2.07	1.81	1.92	1.87

Table S2 Continued.

	3D ED			SCXRD		
**Atom label**	**IAM**	**TAAM-ligand**	**TAAM-ligand+Fe^{III}**	**IAM**	**TAAM-ligand**	**TAAM-ligand+Fe^{III}**
C010	2.33	1.93	1.99	1.49	1.74	1.61
C011	2.14	1.77	1.74	1.65	1.73	1.70
C012	3.44	2.12	2.17	1.41	1.56	1.47
C013	2.14	1.96	2.06	1.37	1.60	1.52
C014	5.69	3.13	3.27	1.98	2.11	2.05
C015	1.72	1.52	1.56	1.60	1.66	1.63
**Average**	2.23	1.79	1.85	1.48	1.60	1.52
**RMSD***	1.08	0.40	0.47	0.08	0.09	N/A

* Root mean square deviations of the R1/R3 ratios for each refinement. The model refined against SCXRD with TAAM-ligand+Fe^{III} scattering factors was used as the reference model for the RMSD calculations

S3. Comparing Spherical and Aspherical Contribution of the Atomic Scattering Factors

The independent atom model (IAM) views atoms as spherical, whereas the transferable aspherical atom model (TAAM) includes aspherical contributions, modelled by higher multipole moments in the multipolar model. TAAM scattering factors comprise both spherical and aspherical parts. The aspherical component can be derived by subtracting the spherical part from the total TAAM scattering factor. When considering aspherical contributions, atomic scattering factors becomes a complex number, and the aspherical contribution varies along different directions. To quantify spherical and aspherical contributions, we calculated the modulus |f| of appropriate scattering factors. The moduli for spherical contributions to the TAAM-ligand+Fe^{III} model are annotated as |f|(Sph-Fe^{III}) for Fe and |f|(Sph-O^{-}) for O, and the total TAAM scattering factors are represted by |f|(Fe^{III}) and |f|(O^{-}) . It's noteworthy that despite a positive modulus, the TAAM-ligand+Fe^{III} electron scattering factor for oxygen in the low-resolution range is negative due to its negative charge. For Fe, the analysis was focused along the Fe-O bond, while for O, two directions were considered: Fe-O (O>Fe) and C=O (O>C). Comparisons were made with the IAM modulus for neutral atoms, annotated as |f|(Fe^{0}) for Fe and |f|(O^{0}) for O.

To quantify the aspherical contributions to the total TAAM scattering factors, the differences in moduli between total and spherical contributions were calculated at chosen directions. Note that the difference between the moduli will be smaller than the modulus of actual difference between the two complex numbers representing the total TAAM scattering factor and spherical contribution to it. Nevertheless, to simplify the illustration the absolute and relative differences between various scattering vectors, we chose to use the difference between the moduli. Furthermore, these moduli were compared to those of IAM for neutral species, assessing the impact of valency (both partial charge and asphericity). This comparison elucidates how differences between IAM and TAAM scattering factors stem from asphericity only (total TAAM minus spherical TAAM) and from accounting for correct valency (total TAAM minus IAM).

Figures S1a and d show the resulting moduli for the total TAAM scattering factor for Fe (|f|(Fe^{III})), the moduli of the spherical contribution |f|(Sph-Fe^{III}) to it and the moduli from the IAM scattering factor for neutral Fe (|f|(Fe^0)) for electron and X-ray scattering, respectively. As shown in Figures S1b and c, the spherical contribution to the total TAAM electron scattering factors for Fe is small, peaking at only 0.35% within the 0.54-0.47 $Å^{-1}$ range (equivalent to 0.94-1.08 Å resolution). This implies that Fe is accurately represented by the spherical model, with asphericity having negligible impact on its atomic scattering factor. The importance of accounting for correct valencies (partial charge and apshericity) is highlighted in Figure S1b, where the difference between |f|(Fe^{III}) and |f|(Fe^0) is shown. The contribution of correct valencies is substantial, amounting to 10% at a resolution of 0.095 $Å^{-1}$ (5.26 Å), and increases further at lower resolutions. For X-ray scattering factors (Figures S1 e and f), the asphericity of iron is most significant at higher resolution ranges, with its maximum impact being 0.56% at 0.64-0.71 $Å^{-1}$ (0.70-0.79 Å resolution). Conversely, the influence of correct valency (partial charge and asphericity together) is more pronounced in the lower resolution range, reaching up to 1.1% between 0-0.10 $Å^{-1}$ (up to d=5.00 Å). Thus, for X-ray scattering, the effects of asphericity and correct valency are comparable, but this is not the case for electron scattering. For electron scattering of Fe, the contribution from partial charge dominates over the contributions of aspherical components of the scattering factor, at least as viewed along the Fe-O bond direction.

For oxygen, as shown in Figure S2, the aspherical contribution to the total TAAM scattering factors (|f|(O^->Fe) and |f|(O^->C)) is significantly higher than for Fe. The aspherical contribution to the total TAAM scattering factor in electron scattering peaks at 65.9% in the O>C direction and 31.92% in the O>Fe direction at 0.045 $Å^{-1}$ (d=11.11 Å). Below 0.02 $Å^{-1}$ (d=25 Å) and above 0.07 $Å^{-1}$ (d=7.14 Å), the aspherical contribution in the O>C direction is under 1%, and likewise in the O>Fe direction below 0.035 $Å^{-1}$ (d=14.29 Å) and above 0.075 $Å^{-1}$ (d=6.67 Å). However, these contributions are still considerably less than those arising from correct valences (partial charge and apshericity together), as shown in Figure S2b. The differences between the moduli of |f|(O^->Fe) or |f|(O^->C) and |f|(O^0) are as high as 778% and 339% respectively at 0.045 Å-1 (11.11 Å). This demonstrates that the major portion

of the difference between TAAM and IAM models in electron scattering stems from accounting for the correct partial charge of oxygen, rather than asphericity alone.

In case of X-ray scattering, similar to Fe, the impact of oxygen asphericity is more pronounced at resolutions above 0.04 Å-1 (d=12.5 Å), reaching a maximum of 2.6% at 0.505-0.620 Å-1 (d=0.81-0.99 Å) in the O>C direction and 1.0% in the O>Fe direction. The contribution from valencies is dominant below 0.04 Å-1 (12.5 Å), with the difference between the moduli of the TAAM scattering factors (|f|(O^->Fe) and |f|(O^->C)) and the IAM factor for neutral oxygen (|f|(O^0)) peaking at 2.8% at 0.065-0.140 Å-1 (3.57-7.69 Å) in the O>Fe direction and 3.1% at 0.125-0.270 Å-1 (d=1.85-4.00 Å) in the O>C direction. As for iron X-ray scattering factors, the contributions from correct valencies and asphericity are of a similar magnitude but affect different resolution ranges.

Overall, this data indicates that for electron scattering factors, the impact of accounting for the correct partial charge prominently dominates the characteristics of the TAAM scattering factors. For X-rays, the effects of correct partial charge and asphericity on the TAAM scattering factors are similar, with asphericity being more crucial at higher resolution ranges, while correct partial charges are more significant at lower resolutions.

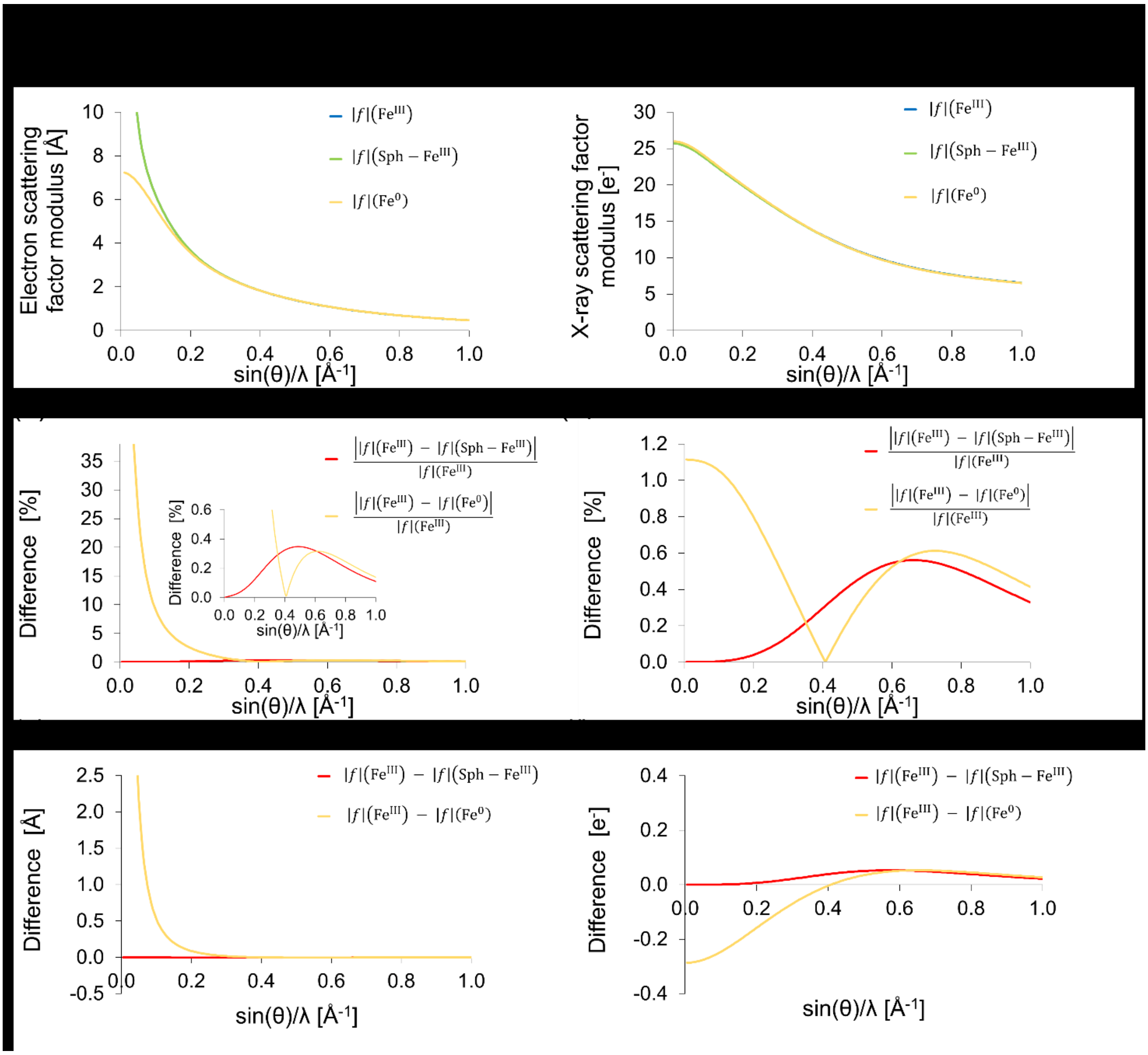

Figure S1 Moduli of atomic scattering factors for electrons (a) and X-rays (b), showcasing the total TAAM scattering factor (|f|(Fe^{III})), its spherical component (|f|(Sph-Fe^{III})), and the IAM equivalent (|f|(Fe^{0})). The figure also includes the percentage difference in moduli relative to the total TAAM factor for both electrons (b) and X-rays (e), along with the absolute differences in moduli for electrons (c) and X-rays (f).

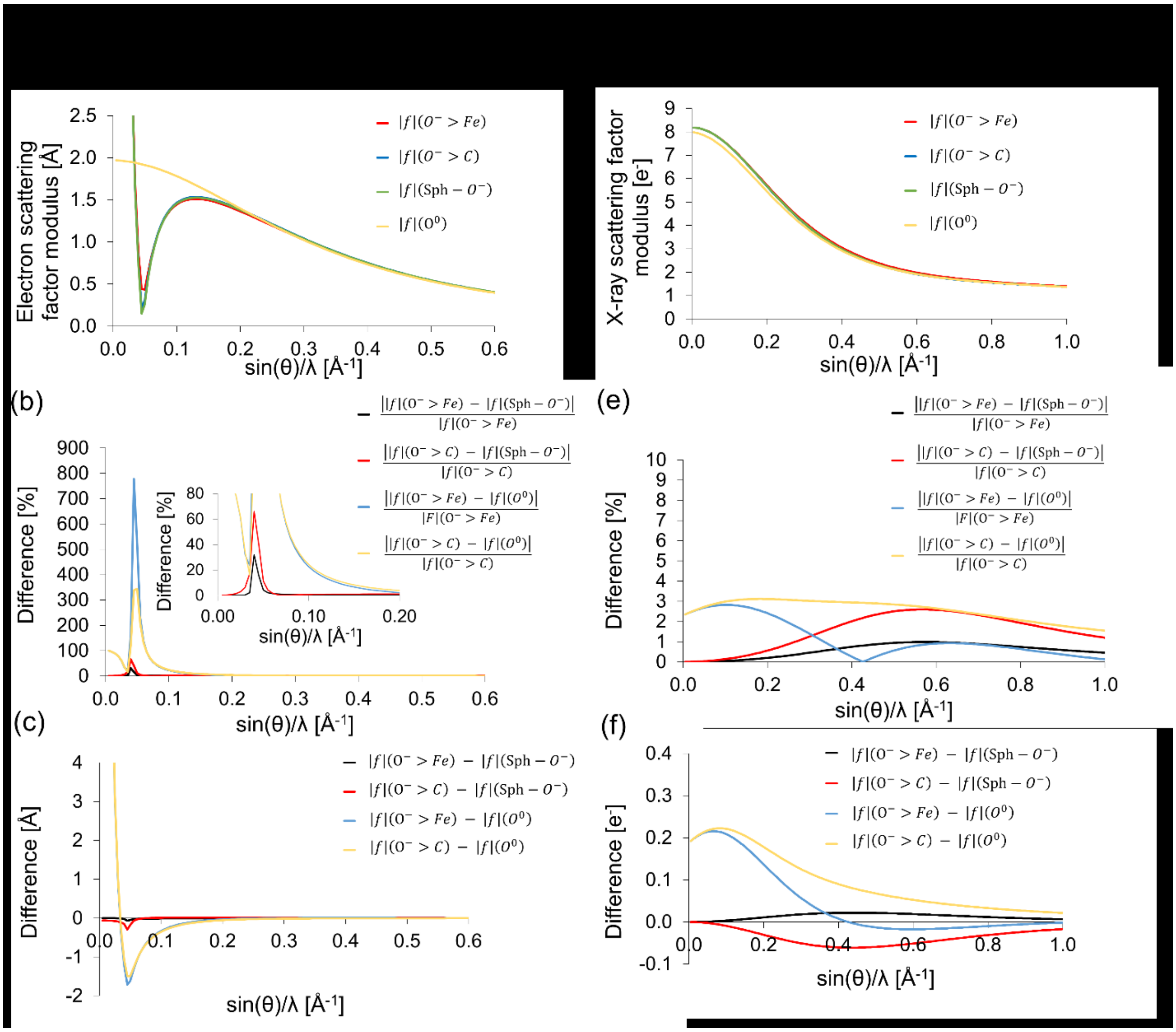

Figure S2 Moduli of the atomic scattering factors for electrons (a) and X-rays (d) showcasing the total TAAM scattering factor in the direction along the O-Fe bond (|f|(O^->Fe)) and the O=C bond (|f|(O^->C)), the spherical contribution to the TAAM scattering factor (|f|(Sph-O^-)) and the IAM equivalent spherical and neutral atomic scattering factor (|f|(O^0)). The figure also includes the percentage difference in moduli relative to the total TAAM factor for both electrons (b) and X-rays (e), along with the absolute differences in moduli for electrons (c) and X-rays (f).

S4. Parametrization of Charged Electron Atomic Scattering Factors Based on IAM

Table S3 4-Gassian parametrized charged electron scattering factors based on IAM used in the refinements against 3D ED data.

Element	a1, b1	a2, b2	a3, b3	a4, b4	RMSE
Fe^{2+}*	43.687, 581.141	1.292, 0.861	3.029, 9.421	9.662, 106.746	0.065
Fe^{3+}*	67.162, 618.91	1.603, 1.091	3.65, 13.661	15.701, 123.602	0.082
$O^{-0.5}$**	-16.477, 916.129	-3.904, 177.365	1.068, 5.245	0.246, 0.536	0.008

* Scattering factors and respective RMSE obtained from the UCLA 4-gaussian fitting (Saha et al., 2022) which are fitted to the charged scattering factors listed in International Tables for Crystallography Vol. C (Cowley et al., 2006).

** The scattering factor and estimated using Yonekura and Maki-Yonekura's method (Yonekura & Maki-Yonekura, 2016) and fitted with a 4-Gaussian curve via nonlinear least-squares optimization in Python using the Levenberg-Marquardt algorithm, available through Edtools (Smeets et al., 2022).
